# Trends of Microorganisms and Antibiotic Resistance Isolated from Patients with Bacterial Keratitis from a Tertiary Hospital in Southeastern Korea: A 26-Year Retrospective Medical Record Review

**DOI:** 10.3390/antibiotics15020207

**Published:** 2026-02-13

**Authors:** Chan-Ho Cho, Jong Ho Lee, Sang-Bumm Lee

**Affiliations:** 1 Department of Ophthalmology, College of Medicine, Yeungnam University, 170 Hyeonchung-ro, Nam-gu, Daegu 42415, Republic of Korea; blitz527@naver.com; 2 Department of Laboratory Medicine, College of Medicine, Yeungnam University, 170 Hyeonchung-ro, Nam-gu, Daegu 42415, Republic of Korea; leejongho@ynu.ac.kr

**Keywords:** *Acinetobacter*, antibacterial agents, corneal ulcer, drug resistance, vancomycin-resistant *Enterococci*

## Abstract

Background. The aim of this study is to analyze changing trends in isolated organisms and antibiotic resistance of bacterial keratitis (BK) over 26 years. Methods. A retrospective medical record review included 542 strains isolated from 462 BK patients between 1998 and 2023. We analyzed routinely generated in vitro antibiotic susceptibility testing results recorded in the laboratory information system and did not perform additional susceptibility testing for research purposes. The entire period was divided into two (first half: 1998–2010, 297 isolates from 255 patients; second half: 2011–2023, 245 isolates from 207 patients) and compared. Results. During the entire period, *Staphylococcus* spp. (32.3%) and *Pseudomonas* spp. (18.1%) were common isolates, and a significant increase in *Acinetobacter* spp. (1.3% vs. 10.6%, *p* < 0.001) was observed. Among Gram-positive bacteria, methicillin resistance rates remained stable between the two periods (52.6% vs. 46.7%, *p* = 0.525), and an increase in vancomycin-resistant *Enterococci* (VRE, 0% vs. 26.1%, *p* = 0.074) was found. Among Gram-negative bacteria (GNB), ciprofloxacin (7.5% vs. 14.4%, *p* = 0.108) and imipenem (2.9% vs. 6.5%, *p* = 0.255) resistance increased slightly, resistance to ceftazidime (8.3% vs. 8.8%, *p* > 0.999) was maintained, and resistance to aminoglycosides (17.8% vs. 7.2%, *p* = 0.010) decreased. Conclusions. Our study suggests that conventional topical fortified antibiotic eye drops (tobramycin, ceftazidime) can still be considered as an empirical treatment option for BK. However, our findings revealed a long-term trend of increasing *Acinetobacter* spp. and VRE, as well as a slight trend of increasing resistance to ciprofloxacin and imipenem in GNB, which may present future challenges in BK treatment.

## 1. Introduction

Causative pathogens of infectious keratitis include bacteria, fungi, viruses, and acanthamoeba, among which bacteria are the leading cause [1]. In infectious keratitis cases, corneal scraping culture tests are performed to identify microorganisms. Afterward, empirical antimicrobial treatment is usually administered before obtaining culture results. In bacterial keratitis (BK), eye drops commonly used for empirical treatment include moxifloxacin, tobramycin, ceftazidime, cefazolin, and vancomycin.

Antibiotic susceptibility results may differ even within the same strain depending on the acquisition of resistance to a specific drug, which plays a significant role in selecting appropriate treatment [2]. Further, antibiotic-resistant strains have been reported in recent decades. In empirical treatment, the possibility of antibiotic resistance may be suspected when there is no drug response, which can sometimes significantly affect treatment outcomes. Therefore, changes in the causative microorganisms responsible for bacterial keratitis, as well as antibiotic resistance trends over time, need to be understood to identify treatment strategies for infectious keratitis.

The emergence of antibiotic-resistant strains warrants further clinical attention [3]. A representative example is the well-known methicillin-resistant staphylococci (MRS) [4]. Recently, multidrug-resistant (MDR) bacteria associated with systemic infections have become an issue. These include MDR *Pseudomonas aeruginosa* (MRPA) [5], MDR *Acinetobacter baumannii* (MRAB) [6], vancomycin-resistant *Enterococci* (VRE) [7], carbapenem-resistant *Enterobacterales* (CRE) [8], and extended-spectrum β-lactamase-producing *Enterobacterales* (ESBL) [9]. Furthermore, these MDR strains have been found in BK [10,11,12,13,14].

The antibiotic resistance rate of strains can differ depending on the type and duration of antibiotic prescriptions in each region and country, environmental transmission, and genetic mutations of the causative microorganisms [15]. The Yeungnam region, located in the southeastern part of Korea, is characterized by many rural areas, a large agricultural population, and a relatively warm climate, unlike the metropolitan areas of Seoul and Gyeonggi that have large urban populations. These characteristics may contribute to differences in the incidence, causative factors, strains, and antibiotic susceptibility of infectious keratitis. Therefore, it is crucial to understand these factors in individual regions within a country. For systemic infections, several studies conducted within the Yeungnam region have reported increasing trends in antibiotic resistance among Gram-negative bacteria [16].

Yeungnam University Hospital, where this study was conducted, is a major tertiary referral hospital located in Daegu Metropolitan City. It provides medical care across a wide area, encompassing both urban and rural areas in the southeastern provinces of Korea, including Daegu, Gyeongsangbuk-do, and Gyeongsangnam-do. The aim of this study was to analyze changing trends in long-term microbial strain distribution and antibiotic resistance in BK in the Yeungnam region through retrospective medical record review. This retrospective study covers the longest study period of 26 years among single-center studies in Korea.

## 2. Results

### 2.1. Enrolled Patients and Annual Distribution of Bacteria

**Figure 1:**
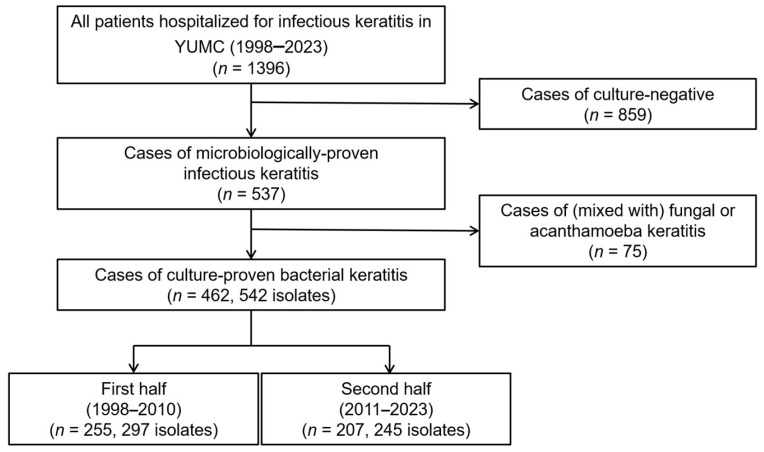
Flowchart of patient enrollment in the study.

During the study period, 1396 patients with infectious keratitis were admitted to Yeungnam University Hospital. Of these patients, 537 were culture-positive (culture positivity rate, 38.5%) after excluding 859 patients with culture-negative results. Subsequently, 75 additional patients with mixed fungal or acanthamoeba keratitis were excluded. Finally, 462 patients (542 isolates) with culture-positive BK were enrolled (Figure 1). Among the total 542 strains, 236 (43.5%) were Gram-positive bacteria (GPB), and 306 (56.5%) were Gram-negative bacteria (GNB).

**Figure 2:**
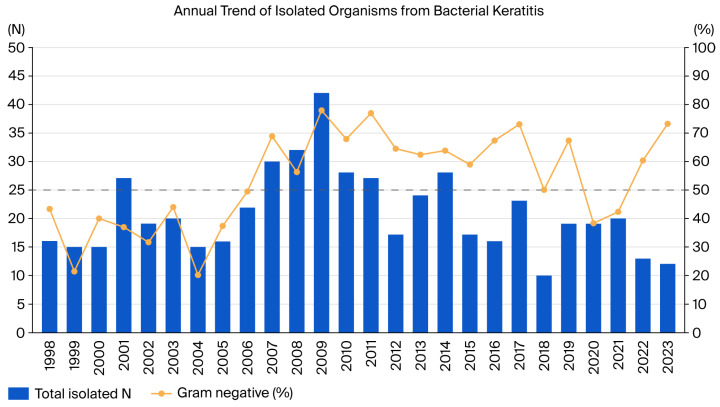
The annual distribution of bacterial isolates in keratitis between 1998 and 2023. Dash line: indicates the point where the proportion of Gram-negative bacteria reaches 50%.

The distribution of the number of patients and strains by period was 255 patients (297 strains) in the first half of the year and 207 patients (245 strains) in the second half. Figure 2 illustrates the annual number of isolated strains from patients with keratitis, the proportion of GNB, and their trends. Prior to 2006, the proportion of GPB was higher than that of GNB, and from 2007 onward, except for 2020–2021, the proportion of GNB was more than half (Figure 2). Polymicrobial infection cases were identified in 67 of 462 individuals (14.5%), with 56 cases containing two strains, 9 cases containing three strains, and 2 cases containing four strains.

### 2.2. Microbiological Profiles and Trends of Gram-Positive and -Negative Isolates

The microbiological profile of bacterial isolates throughout the entire period was investigated. The most common species in GPB and GNB were *Staphylococcus* spp. (175 strains, 32.3%) and *Pseudomonas* spp. (98 strains, 18.1%), respectively. Comparing the first and second halves, the proportion of total GPB (48.8% vs. 37.1%, *p* = 0.007) and *Staphylococcus* spp. (38.7% vs. 24.5%, *p* < 0.001) decreased, whereas the proportion of *Enterococcus* spp. increased (4.0% vs. 9.4%, *p* = 0.014). The proportion of total GNB (51.2% vs. 62.9%, *p* = 0.007), *Acinetobacter* spp. (1.3% vs. 10.6%, *p* < 0.001), *Achromobacter* spp. (1.3% vs. 6.5%, *p* = 0.002), and *Leclercia* spp. (0% vs. 3.3%, *p* = 0.002) increased. The proportions of *Pseudomonas* spp., *Enterobacter* spp., and *Serratia* spp. demonstrated no significant differences between the two periods (Table 1). The species-level frequencies for all isolated genera are provided in Supplementary Table S1.

### 2.3. Demographic Data

Table 2 presents the demographic data for all 462 patients between the two study periods. The mean age was significantly higher in the second half (57.3 years) than in the first half (48.7 years) (*p* < 0.001). There were no significant differences in male sex (53.3% in the first half, 56.0% in the second half, *p* = 0.574), mean symptom duration (8.3 days vs. 10.1 days, *p* = 0.339), contact lens wear (21.6% vs. 19.3%, *p* = 0.565), prior use of topical antibiotics (48.2% vs. 49.8%, *p* = 0.779), prior use of topical steroids (13.7% vs. 15.5%, *p* = 0.598), or prior ocular surface disease (30.2% vs. 26.6%, *p* = 0.409) between the two periods.

The prevalence of diabetes was slightly higher in the second half (8.2% vs. 12.1%, *p* = 0.211), but the difference was not significant. Prior ocular surgery was significantly higher in the second half (17.3% vs. 27.5%, *p* = 0.009). The proportion of patients with agricultural occupations slightly increased (20.0% vs. 27.5%, *p* = 0.061) (Table 2).

### 2.4. In Vitro Antibiotic Resistance Trends of GPB

Throughout the entire period, GPB exhibited low antibiotic resistance to linezolid (0%), tigecycline (1.2%), rifampicin (3.4%), and vancomycin (3.5%). Further, they showed relatively low antibiotic resistance to cefotaxime (8.3%), nitrofurantoin (11.8%), moxifloxacin (12%), and synercid (12.4%), and relatively high resistance to ciprofloxacin (35.4%), imipenem (36.7%), levofloxacin (46.3%), and oxacillin (50.6%) (Table 3).

Comparing the first and second halves, GPB demonstrated a significant increase in resistance to vancomycin (0.7% vs. 7.8%, *p* = 0.007) and nitrofurantoin (0% vs. 15.9%, *p* = 0.036). However, resistance to gentamicin (52.6% vs. 34.4%, *p* = 0.026) decreased in GPB. No significant changes in resistance to fluoroquinolones were observed in overall GPB (Table 3).

**Figure 3:**
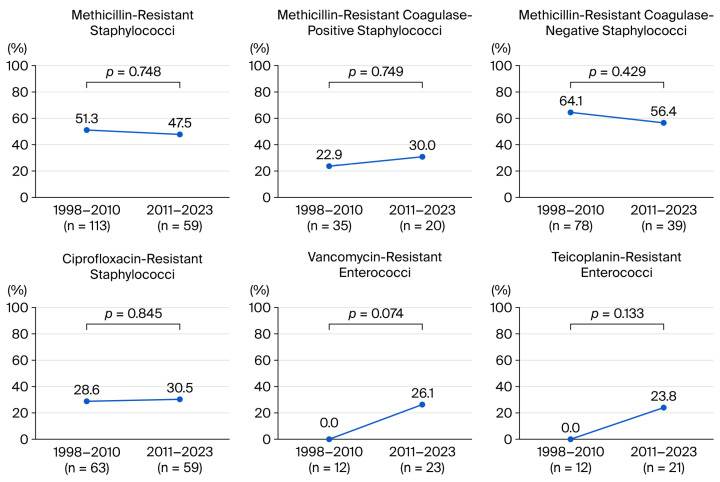
Antibiotic resistance of representative Gram-positive species over 26 years.

Among the resistant strains, the proportion of MRS (51.3% vs. 47.5%, *p* = 0.748), methicillin-resistant coagulase-negative staphylococci (MRCoNS, 64.1% vs. 56.4%, *p* = 0.429), and methicillin-resistant coagulase-positive staphylococci (MRCoPS, 22.9% vs. 30%, *p* = 0.749) remained stable during the study period. In *Enterococcus* spp., teicoplanin-resistant *Enterococcus* spp. (0% vs. 23.8%, *p* = 0.133) and VRE (0% vs. 26.1%, *p* = 0.074) exhibited an increasing trend in the second half. In *Streptococcus* spp., no significant change was found in the antibiotic resistance trend (Figure 3) (see Supplementary Table S2).

### 2.5. In Vitro Antibiotic Resistance Trends of GNB

Throughout the entire period, GNB demonstrated low antibiotic resistance to ertapenem (2.6%), meropenem (3.7%), imipenem (4.7%), cefepime (5.3%), amikacin (6.3%), levofloxacin (7.0%), gentamicin (8.2%), ceftazidime (8.6%), and colistin (8.7%). Further, they showed relatively low antibiotic resistance to tobramycin (10.9%) and ciprofloxacin (11.1%), with relatively high resistance to tigecycline (26.7%), trimethoprim/sulfamethoxazole (29.5%), and ticarcillin (34.3%) (Table 4).

Comparing the first and second half, GNB demonstrated a significant increase in resistance to aztreonam (26.4% vs. 53.1%, *p* < 0.001) and piperacillin (18.3% vs. 32.7%, *p* = 0.018) and slightly increased resistance to ciprofloxacin (7.5% vs. 14.4%, *p* = 0.108) and imipenem (2.9% vs. 6.5%, *p* = 0.255). In addition, resistance to ceftazidime (8.3% vs. 8.8%, *p* > 0.999) was maintained, while antibiotic resistance to ampicillin (97.2% vs. 81.0%, *p* = 0.005), amikacin (10.5% vs. 1.6%, *p* = 0.004), and gentamicin (14.2% vs. 2.9%, *p* = 0.001), and aminoglycosides (17.8% vs. 7.2%, *p* = 0.010) exhibited a decreasing trend (Table 4).

**Figure 4:**
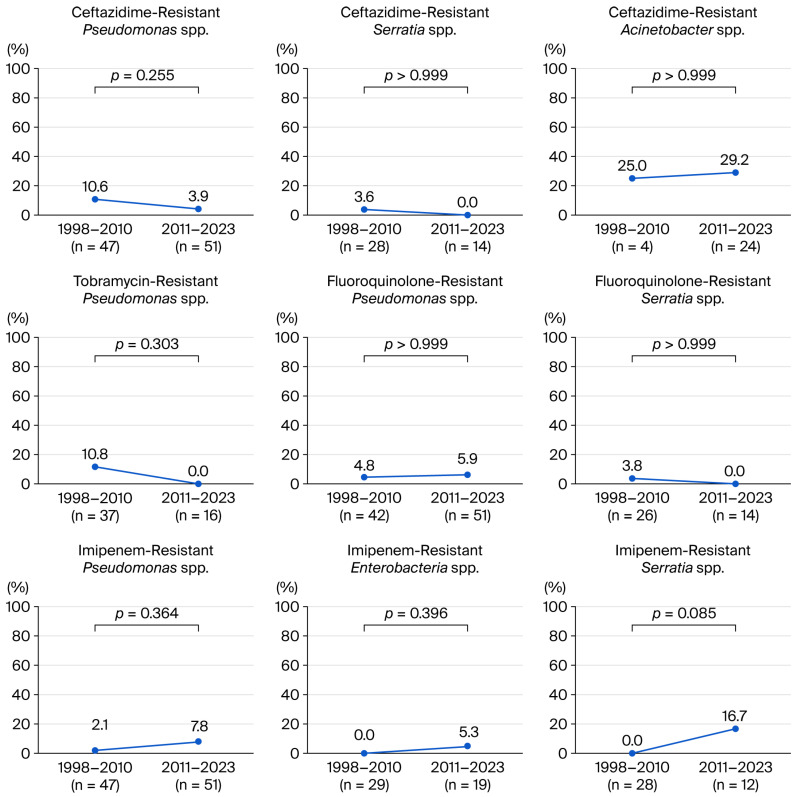
Antibiotic resistance of representative Gram-negative species over 26 years.

In *Pseudomonas* spp., resistance to aztreonam (42.1% vs. 70.6%, *p* = 0.009) and ticarcillin (27.7% vs. 64.3%, *p* = 0.024) significantly increased, whereas resistance to amikacin (10.6% vs. 0%, *p* = 0.024) and gentamicin (14.9% vs. 2.0%, *p* = 0.026) decreased. Resistance to ceftazidime (10.6% vs. 3.9%, *p* = 0.255) and tobramycin (10.8% vs. 0%, *p* = 0.303) slightly decreased in *Pseudomonas* spp. (Figure 4) (see Supplementary Table S3). In *Serratia* spp., resistance to cefoxitin (33.3% vs. 69.2%, *p* = 0.070) increased in the second half, while no significant change in resistance trends was found for other antibiotics. In *Enterobacter* spp., no significant change was found in antibiotic resistance trends. In *Acinetobacter* spp., resistance to ceftazidime (25.0% and 29.2%) was higher than in other GNB in both periods.

Regarding fluoroquinolone resistance, there was no significant change in *Pseudomonas* (4.8% vs. 5.9%, *p* > 0.999) or *Serratia* spp. (3.8% vs. 0%, *p* > 0.999) (Figure 4) (see Supplementary Table S3). Regarding carbapenem resistance in GNB, a slight increasing trend in resistance to imipenem was observed over time (*Pseudomonas* spp.: 2.1% vs. 7.8%; *Enterobacter* spp.: 0% vs. 5.3%; *Serratia* spp.: 0% vs. 14.3%; *Acinetobacter* spp.: 0% vs. 3.8%) but was not statistically significant (Figure 4) (see Supplementary Table S3).

### 2.6. MDR Strains

The analysis of the frequency of MDR bacteria in keratitis over 26 years identified two ESBL strains (one in the first half and one in the second half), six VRE strains (none and six), five CRE strains (one and four), and one MRAB strain (none and one). No MRPA was identified during the study.

## 3. Discussion

The main results of this retrospective study are as follows: (1) The proportion of GNB was high at over 50% in both the first and second halves, and GNB demonstrated a significant increase in the second half. (2) The two most common species throughout the entire period were *Staphylococcus* spp. and *Pseudomonas* spp. (3) *Staphylococcus* spp. demonstrated a decreasing trend over time, whereas *Enterococcus* spp. and *Acinetobacter* spp. showed an increasing trend. (4) In the antibiotic resistance of GPB, the proportion of MRS remained stable at 50% with no change in trend, whereas VRE showed an increasing trend. (5) In GNB, ceftazidime resistance remained stable at less than 10% during the study period, and aminoglycoside resistance decreased. In contrast, ciprofloxacin and imipenem resistance showed a slight increasing trend in GNB.

The culture positivity rate in this study was 38.5%. Zhang et al. reported a global meta-analysis (including 38 published articles between 2000 and 2020) of the pathogens and antibiotic susceptibilities of BK, revealing an average culture positivity rate of 47% (95% confidence interval [CI]: 42–52%) [15]. *Staphylococcus* spp. were the most common species in both periods in this study, with a decreasing trend over time. *Staphylococcus* spp. are the most common species in BK on all continents globally, and *Pseudomonas* spp. are also commonly reported [15]. In a Korean study, Mun et al. reported that the most common bacterial species in infectious keratitis between 2007 and 2016 were coagulase-negative staphylococci (15.9%), followed by *S. aureus* (12.1%) and *P. aeruginosa* (10.3%), with comparable results to the trend found in this study [17]. Strain distribution may vary across geographic regions, and analyzing strain distribution within a region provides useful guidance for empirical treatment. In our region, GNB were predominant and exhibited an increasing trend. A study in the United Kingdom reported an increased rate of GNB, which is similar to our result [18]. Conversely, several studies have reported a predominance of GPB in the strain distribution [15].

*Acinetobacter* spp. demonstrated an increasing trend in our region. Interestingly, no studies reported similar trends in neighboring regions or other countries. Globally, *Acinetobacter* spp. keratitis is rarely reported [19,20,21]. *Acinetobacter* spp. are popularly associated with nosocomial infections, primarily occurring in compromised conditions, and are known to have a high rate of antibiotic resistance [22]. In this study, *Acinetobacter* spp. showed higher resistance to ceftazidime (28.6%) compared with other GNB. The overall prevalence of MDR strains in patients with *A. baumannii* based on systemic infection criteria is estimated to be 79.9%, but this has not been investigated in the ophthalmological field [23]. Therefore, the increased presence of this strain in keratitis warrants clinical attention. The authors are currently conducting a follow-up study that compares the clinical features and treatment outcomes between *A. baumannii* and *P. aeruginosa* strains in keratitis. The results analyzed to date reveal that the surgical treatment rate (23.5% vs. 14.0%, *p* = 0.448) and poor treatment outcome rate (56.2% vs. 30.8%, *p* = 0.077) were higher in the *A. baumannii* group [24].

The emergence of antibiotic-resistant bacteria in infectious keratitis may affect the response to antibiotic eye drops. Broad-spectrum antibiotics (levofloxacin, moxifloxacin, cefazolin, vancomycin, tobramycin, and ceftazidime) are commonly used empirically for BK. Therefore, the increase in vancomycin resistance of GPB (0.7% vs. 7.8%), including VRE (0% vs. 26.1%), in our study is noteworthy.

In this study, 50% of staphylococci were MRS. The Antimicrobial Resistance Monitoring of Ocular Microbiota in the United States (ARMOR) study reported a methicillin-resistant *Staphylococcus aureus* (MRSA) rate of 36.6% [25]. In a previous Korean multicenter study, the prevalence of methicillin-resistant *Staphylococcus epidermidis* was 64%, higher than the 47% reported for MRSA; other studies reported the proportion of MRSA among *S. aureus* keratitis at approximately 25–44% [26,27] and MRCoNS among coagulase-negative staphylococci at approximately 19–62% [28,29,30]. This was similar to the trend of MRCoPS at 25.5% and MRCoNS at 61.5% in this study. Meanwhile, studies revealed differences depending on the country and region where the studies were conducted, such as in a Swedish study, where MRSA was not detected at all, and in one conducted in Toronto, where MRSA was detected in only 1.3% of cases [28,31].

Regarding fluoroquinolones in GPB, Alexandrakis et al. reported that ciprofloxacin resistance in *S. aureus* (11% in 1990 and 28% in 1998) was increasing [32]. In this study, ciprofloxacin resistance in GPB was 35.4% (30.3% in the first half vs. 39.5% in the second half), and the ciprofloxacin resistance in *Staphylococcus* spp. was 29.5% (28.6% vs. 30.5%). Further, the levofloxacin resistance of GPB was high (46.3%) throughout the entire period, with a slight increase from 40% in the first half to 51.7% in the second half. In the case of moxifloxacin, the number of tests in the second half (*n* = 4) was much smaller than in the first half (*n* = 21), so there is insufficient evidence to conclude that the resistance rate increased in the second half. Nevertheless, moxifloxacin showed a relatively good susceptibility rate of 88% in GPB throughout the entire study period. In comparison, a meta-analysis reported that moxifloxacin sensitivity in GPB after 2010 was between 60% and 85%, which was slightly lower than the results of this study [15]. Antibiotic resistance is frequently reported for moxifloxacin, and the drug resistance rate may increase when moxifloxacin monotherapy is performed. The ARMOR study reported that prior exposure to topical fluoroquinolones is a potential factor contributing to resistant strain selection [25]. Data from a randomized controlled trial (the Steroids for Corneal Ulcers Trial) showed that a twofold elevation in the MIC of moxifloxacin was associated with worse treatment outcomes, including worse visual acuity and increased infiltrate size after 3 weeks of treatment, and slower time to reepithelialization [33].

Vancomycin is a powerful glycopeptide antibiotic administered to treat GPB infections resistant to other antibiotics. Most previous studies reported that vancomycin susceptibility has been close to 100% in BK, with resistance reported only in a few cases. Therefore, the increasing trend of VRE in this study warrants clinical attention. To date, no studies have reported an increase in VRE in patients with keratitis. VRE can be divided into low-level (VanC gene: *E. gallinarum*, *E. casseliflavus*) and high-level resistance (VanA, VanB, VanD gene: *E. faecium*) based on genotype [34]. Of the six VRE strains in this study, five were *E. faecium* (high-level resistance). In VRE infection cases, linezolid can be administered for treatment, and 0.2% linezolid eye drops may be useful in VRE keratitis [12]. The linezolid susceptibility in this study was 100%, and two cases of successful treatment with topical 0.2% linezolid in patients with VRE during the study period were reported.

In GNB, ciprofloxacin resistance and imipenem resistance showed a slightly increasing trend, whereas tobramycin and ceftazidime exhibited good susceptibility throughout the study period, with no significant change in susceptibility over time. Previously, Alexandrakis et al. revealed that ciprofloxacin resistance in *P. aeruginosa* was approximately 0–1% with no increasing trend [32]. In this study, the ciprofloxacin resistance of *Pseudomonas* spp. was slightly higher in the second half (4.8% vs. 5.9%), and the ciprofloxacin resistance of all GNB also showed an increasing trend (7.5% vs. 14.4%), which is clinically noteworthy. A global meta-analysis study reported that the resistance of GNB to ciprofloxacin was less than 10% [15]. A previous Korean study demonstrated similar results to this study, reporting ciprofloxacin resistance of GNB at 8.8% [17].

Imipenem is a β-lactam antibiotic that belongs to the carbapenem family. It has broad-spectrum properties and is effective against aerobic and anaerobic GPB and GNB, including *Pseudomonas aeruginosa* and *Enterococcus*. The mechanism of its bactericidal effect is the inhibition of bacterial cell wall synthesis [35]. To the best of our knowledge, only a few studies have reported on the prevalence of imipenem resistance in BK. In MRPA keratitis, Vazirani et al. reported that most antibiotics were resistant, with only colistin and imipenem exhibiting resistance in 56.52% of cases, indicating the possibility of using imipenem for infections caused by resistant bacteria that do not respond to conventional treatment [10]. Our study did not demonstrate statistically significant changes in GNB resistance to imipenem, whereas a slight increase in resistance was observed in all major GNB species, indicating that future trends should be closely monitored.

Aztreonam is also a β-lactam antibiotic of the monobactam family, effective against GNB. This study observed a significant increase in aztreonam resistance, particularly in *P. aeruginosa*. While aztreonam eye drop has been rarely been used for the treatment of keratitis [36], the increasing trend in resistance suggests the need for close monitoring.

The aminoglycoside resistance in GNB in this study was 6.3–10.9%, whereas other studies, including in Auckland, New Zealand (0%) [37], Nottingham, UK (1.3–1.9%) [38], eastern England (2.5%) [39], and Germany (4%) [40], reported lower rates than in this study, and Taiwan (5.6–9.4%) reported similar findings [41]. Resistance to ceftazidime in GNB was 8.6%, which was similar to global meta-analyses (0–10%) [15] and a study in Toronto (8.6%) [42], whereas higher rates were reported in north-central India (approximately 30%) [43,44], and lower rates were reported in Auckland (0%) [37], Seoul (0%) [17], St. Louis (0%) [45], and Taiwan (0.4%) [41].

Interestingly, this study revealed an overall increasing trend in resistance to vancomycin and imipenem, whereas in GNB, resistance to ceftazidime remained constant, and resistance to aminoglycosides even decreased. This trend may be related to global trends in antibiotic use and resistance. Studies investigating global trends in systemic antibiotic consumption and the identification of resistant bacteria found that the use of broad-spectrum antibiotics such as BLBLI, carbapenem, and vancomycin has increased, and resistance to these antibiotics has also increased [46,47,48,49,50]. In contrast, traditionally used antibiotics such as ceftazidime and aminoglycosides have shown a recent trend toward decreasing use [46]. The results of this study are similar to these global trends in antibiotic resistance [46]. Based on the results of this study, topical antibiotics (tobramycin, ceftazidime), currently used primarily for the treatment of bacterial keratitis, can still be considered as empirical treatment options.

Regarding MDR bacteria, studies on ESBL-related keratitis have been rarely reported [11,51]. In this study, ESBL cases included one case of *E. coli* and one of *Klebsiella* spp. Carbapenems (imipenem or meropenem) can be used as a treatment for ESBL infection cases [52]. MRAB has become an issue due to the recent increase in healthcare-associated infections [53]. One case of MRAB in this study reported a history of tree branch trauma and topical steroid use before presentation and was not associated with healthcare-related infection. The case was treated with moxifloxacin and ceftazidime without complications. In another report from Korea, MRAB was isolated in intraocular content cultures, including vitreous and uvea samples, after the evisceration of the eye due to keratitis treatment failure [54]. In the case of MRAB, ophthalmic infections do not respond well to conventional medications, but cefiderocol, colistin, sulbactam–durlobactam, tigecycline, and minocycline may be effective [55]. CRE is primarily associated with systemic and nosocomial infections [56]. In this study, four of the five cases in which CRE was detected were contact lens-related BK, and one case was associated with vegetative corneal trauma, not directly associated with systemic infection. The available drugs for CRE include newer antibiotics, particularly β-lactam–β-lactamase inhibitor combinations (BLBLI, e.g., ceftazidime–avibactam, meropenem–vaborbactam) [57,58]. However, all patients with CRE in this study improved with conventional antibiotic treatment. This may be associated with good susceptibility to conventional topical antibiotics (aminoglycosides, fluoroquinolones, etc.) in all five cases.

Compared to the first half, the second half of this study included more VRE, CRE, *Acinetobacter* spp. (including MRAB), and ciprofloxacin-resistant GNB. MDR strains may be primarily associated with systemic or healthcare-associated infections, and the effect of MDR strains on actual antibiotic treatment outcomes in keratitis remains unclear, thereby requiring further research [3]. However, antibiotic resistance may be associated with antibiotic overprescribing and overuse; thus, caution should be taken when prescribing topical antibiotics in clinical settings. Notably, this study collected data from a tertiary referral hospital, and most cases were referred after already receiving topical antibiotic treatment.

This study provides clinically useful implications, but several limitations should be considered. First, the microbiological workflow (culture media and incubation, identification platforms, AST panels, etc.) changed over the 26-year period; although we addressed this by analyzing contemporaneous categorical interpretations recorded in the laboratory information system and avoiding retrospective breakpoint re-interpretation, residual heterogeneity across eras is unavoidable in a retrospective study. Second, the NCCLS does not have regulations for topical ophthalmic antibiotics; laboratories use serum concentrations, which are significantly lower than concentrations used in eye drops [59,60]. In clinical practice, frequent topical antibiotic administration or the use of fortified antibiotic eye drops leads to significantly higher antibiotic concentrations than systemic antibiotics, which may cause discrepancies between laboratory results and clinical outcomes [61]. Third, although third- and fourth-generation fluoroquinolones are generally known to have higher antibacterial activity, this study was limited to antibiotic cards used in commercially available automated microbiological analyzers, which did not sufficiently perform third- and fourth-generation fluoroquinolone antibiotic susceptibility testing for GNB. Fourth, chocolate agar was not routinely included in the keratitis culture sets, which may have reduced the recovery of rare fastidious organisms (e.g., *Haemophilus* spp. or *Neisseria* spp.). Fifth, as shown in Figure 2, the number of specimens collected for retrospective data analysis was limited and subject to annual fluctuations. In this study, insufficient numbers were registered to analyze the variance and distribution of annual resistance rates for specific antibiotics in specific strains. Therefore, analysis could only be conducted on a large scale, spanning two 13-year periods.

The main strength of this study is that it analyzes a long-term perspective on the microbiological and antibiotic resistance trends of bacterial keratitis from a single region, providing baseline data for reference in clinical practice. Collaborating with other institutions and laboratory medicine professionals in the region to conduct a multicenter study to expand the data could yield even more clinically useful results.

## 4. Materials and Methods

### 4.1. Study Design

We conducted a retrospective, consecutive case-series study of BK at Yeungnam University Hospital from January 1998 to December 2023 (a 26-year period). Patients hospitalized for infectious keratitis with culture-proven bacterial infections were included. The exclusion criteria included culture-negative cases, outpatients, and those with a mixture of fungal and acanthamoeba keratitis. Admission decisions were based on keratitis severity, potential vision threat, and the need for intensive topical antimicrobial agents. A single physician (S.-B.L.) identified these criteria. After inpatient treatment, patients were discharged after achieving complete epithelialization with sterilization, without requiring further surgical treatment.

To identify overall trends, the study period was divided equally into two parts of 13 years each (first half: 1998–2010, second half: 2011–2023), and a comparative analysis by period was performed considering the demographic data, the distribution of isolated strains, and antibiotic resistance.

### 4.2. Demographic Data Analysis

We analyzed the baseline demographic factors of the patients enrolled in this study through a retrospective medical record review to identify overall trends. The demographic data analysis included sex, age, symptom duration, contact lens use, diabetes mellitus, prior topical antibiotic use, prior topical steroid use, prior ocular surface disease, prior ocular surgery, and agricultural occupation at the time of initial presentation to our hospital. Symptom duration was defined as the interval between symptom onset and the initial presentation to our hospital. Prior topical antibiotic use was defined as cases in which topical antibiotics were being used at the time of presentation to our hospital, regardless of the duration or type. Prior topical steroid use was defined as cases in which topical steroids were being used at the time of presentation to our hospital, regardless of the duration or type. We analyzed each demographic factor and made a comparison between the two study periods.

### 4.3. Bacterial Culture and Identification

To identify causative bacteria, specimens were collected through corneal scrapings from all patients before empirical antibiotic administration, and smear and culture tests were conducted. Topical anesthesia was induced with 0.5% proparacaine hydrochloride (Alcaine^®^, Alcon, Fort Worth, TX, USA) before obtaining corneal scrapings from all patients using a No.15 Bard-Parker knife (Bard-Parker Co., Danbury, CT, USA). Sterile cotton-tipped swabs were used to simultaneously collect conjunctival swabs from all patients. The scrapings were smeared on glass slides, and Gram staining was performed. To rule out fungal keratitis, 10% potassium hydroxide (KOH) smear specimens were collected from the margins and base of ulcers and placed within a marked area on a glass slide. One drop of 10% KOH was placed on the smear, and a clean coverslip was added. Acid-fast staining was performed only when clinically indicated (e.g., suspected nontuberculous mycobacterial keratitis). Corneal scrapings were inoculated onto a variety of solid and liquid media that supported bacterial and fungal growth. Cultures of contact lenses and their storage solutions were performed only in patients for whom lens specimens were available. Contact lens specimens were obtained by placing the patient’s contact lens in a plain bottle containing 1.5 cc of distilled water using a sterile McPherson forceps, and lens storage fluid specimens were withdrawn using a sterile 3 cc syringe, placed in a plain bottle, and sent to the microbiology laboratory. Cultures from conjunctival swabs and contact lens-related specimens (if available) were considered adjunct; the primary microbiological analyses in this study were based on the corneal scraping culture results.

Routine bacterial culture used 5% sheep blood agar, MacConkey agar, and thioglycolate broth; fungal culture used Sabouraud dextrose agar. Chocolate agar was not routinely included in the keratitis culture set. The media were incubated at 35–37 °C for an appropriate period under the atmospheric conditions required for each medium and examined daily for organism growth. Incubation conditions (including 5% CO_2_) were set when fastidious growth requirements were suspected/needed, but the routine isolation of *Streptococcus* spp. was not specifically performed under 5% CO_2_ conditions. *Streptococcus* isolates were evaluated using conventional phenotypic characterization (colony morphology/hemolysis on blood agar, catalase test) and, when needed, additional tests such as optochin, bile solubility, or Lancefield grouping in conjunction with the automated system. Susceptibility testing for *Streptococcus*/fastidious organisms followed National Committee for Clinical Laboratory Standards and Clinical and Laboratory Standard Institute (NCCLS/CLSI)-recommended conditions (e.g., Mueller–Hinton agar supplemented with 5% sheep blood and incubation under 5% CO_2_ when required).

Bacterial identification was performed using the VITEK platform (VITEK system; BioMérieux-Co, Lyon, France) with appropriate identification cards (VITEK 1/VITEK 2 GN, GP, and YST ID cards) and, from 2019, MALDI-TOF MS (VITEK MS, BioMérieux-Co, Lyon, France) was additionally used when available. NCCLS/CLSI breakpoints, testing platforms, and antibiotic panels have changed over time. For analysis, we used the categorical interpretations recorded in the laboratory information system based on the criteria applied at the time of testing, and we did not retroactively re-interpret historical results using current breakpoints. The evolution of identification methods and platforms across the study period was as follows: 1998: API kit, disk diffusion, and VITEK; 1999: VITEK 2, introduced with disk diffusion used in parallel until 2007; 2011: VITEK 2 plus VITEK 2 XL; 2019: VITEK MS added. Although our laboratory platforms generally provide species-level identification, we summarized the primary analyses at the genus level to ensure longitudinal comparability because the instruments and databases changed over time. Species-level frequencies for all genera are provided in Supplementary Table S1.

### 4.4. In Vitro Antibiotic Susceptibility Testing

In this retrospective study, we analyzed routinely generated in vitro antimicrobial susceptibility testing (AST) results recorded in the laboratory information system and did not perform additional susceptibility testing for research purposes.

AST was performed using appropriate automated AST cards on the VITEK platform and/or Kirby–Bauer disk diffusion, depending on organism group and antibiotic availability by period (without enumerating each historical card panel). For automated testing, minimal inhibitory concentration (MIC) values were recorded when available; for disk diffusion, inhibition zone diameters were measured and interpreted [62]. Antibiotic resistance was identified following the NCCLS criteria [59]. The disk diffusion method used Mueller–Hinton agar medium, and the inoculum amount of the strain was adjusted following the NCCLS recommendations [59]. An antibacterial disk was placed on the agar evenly inoculated with the bacterial solution, and the inhibition zone diameter was measured to identify the resistance pattern of the strain to the test antibacterial agent. Antibiotic resistance was evaluated using an automated microbiological analyzer, and its routine AST cards were interpreted according to contemporaneous NCCLS/CLSI criteria [63].

For staphylococci, methicillin resistance was determined according to contemporaneous NCCLS/CLSI criteria using oxacillin- and/or cefoxitin-based screening, performed via automated AST and/or disk diffusion depending on the period. Accordingly, MRSA and MSSE were classified based on the methicillin (oxacillin/cefoxitin) interpretive category at the time of testing.

### 4.5. Definition of Antibiotic Resistance and Multidrug Resistance

We defined antibiotic susceptibility as “resistant” if the susceptibility was reported as intermediate or resistant based on a retrospective medical record review. MRS (methicillin-resistant staphylococci) were defined as *Staphylococcus* spp. that are resistant to methicillin (oxacillin/cefoxitin). The scope of MDR defined in the analysis of resistant bacteria was investigated for MRPA, MRAB, VRE, CRE, and ESBL, as previously mentioned. The definition of each MDR bacterium is as follows: MRPA is defined as *P. aeruginosa* that is resistant to all three antibiotic classes: (1) carbapenems, (2) aminoglycosides, and (3) fluoroquinolones [64]. MRAB is defined as *A. baumannii* that is resistant to all three antibiotic classes: (1) carbapenems, (2) aminoglycosides, and (3) fluoroquinolones [53]. VRE is defined as *Enterococcus* spp. that are resistant to vancomycin [65]. CRE is defined as *Enterobacterales* that are resistant to carbapenem antibiotics [66]. ESBL is defined as *Enterobacterales* (primarily *E. coli* or *Klebsiella* spp.) that produce β-lactamases (including those that can degrade broad-spectrum cephalosporins and monobactams) [9].

### 4.6. Statistical Analysis

IBM Statistical Package for the Social Sciences Statistics for Windows (version 25.0; IBM Corp., Armonk, NY, USA) was used for data analyses. Fisher’s exact tests were used to assess categorical data. An independent-sample *t*-test was used to compare the mean values. Statistical significance was set at a *p*-value of <0.05. If the number of subjects was extremely small or the sample size imbalance was so severe that statistical interpretation errors were likely to occur in comparative analyses, percentages or statistical values were avoided as far as possible, and the statistical value was marked as “not applicable (NA)”.

## 5. Conclusions

In conclusion, this study revealed an increasing trend of *Acinetobacter* spp. and VRE during a long-term trend of BK in southeastern Korea, indicating the need for clinical attention. During the study period, GNB showed a significant increase in susceptibility to aminoglycosides and maintained good susceptibility to ceftazidime, but showed a slight increase in resistance to ciprofloxacin and imipenem. Therefore, conventional topical fortified antibiotic eye drops (tobramycin, ceftazidime) can still be considered as an empirical treatment option for BK. The results of this study indicate that each medical institution must identify the causative bacteria and their antibiotic susceptibility trends for the appropriate treatment of infectious keratitis cases. Furthermore, appropriate antibiotics should be selected for patients with BK by considering long-term strain distribution and antibiotic susceptibility trends.

## Figures and Tables

**Table 1:** Microbiological profile of bacterial isolates in keratitis between 1998 and 2023.

Bacterial Isolates	1998–2010 *n* (%)	2011–2023 *n* (%)	Total *n* (%)	*p*-Value
Gram-positive				
*Staphylococcus* spp.	115 (38.7)	60 (24.5)	175 (32.3)	<0.001
*Enterococcus* spp.	12 (4.0)	23 (9.4)	35 (6.5)	0.014
*Streptococcus* spp.	18 (6.1)	8 (3.3)	26 (4.8)	0.159
Subtotal	145 (48.8)	91 (37.1)	236 (43.5)	0.007
Gram-negative				
*Pseudomonas* spp.	47 (15.8)	51 (20.8)	98 (18.1)	0.146
*Enterobacter* spp.	30 (10.1)	19 (7.8)	49 (9.0)	0.370
*Serratia* spp.	28 (9.4)	14 (5.7)	42 (7.7)	0.146
*Stenotrophomonas* spp.	17 (5.7)	15 (6.1)	32 (5.9)	0.857
*Acinetobacter* spp.	4 (1.3)	26 (10.6)	30 (5.5)	<0.001
*Achromobacter* spp.	4 (1.3)	16 (6.5)	20 (3.7)	0.002
*Klebsiella* spp.	8 (2.7)	1 (0.4)	9 (1.7)	0.045
*Leclercia* spp.	0 (0.0)	8 (3.3)	8 (1.5)	0.002
*Escherichia* spp.	3 (1.0)	2 (0.8)	5 (0.9)	NA
*Delftia* spp.	3 (1.0)	0 (0.0)	3 (0.6)	NA
*Proteus* spp.	1 (0.3)	1 (0.4)	2 (0.4)	NA
*Morganella* spp.	2 (0.7)	0 (0.0)	2 (0.4)	NA
*Pantoea* spp.	1 (0.3)	0 (0.0)	1 (0.2)	NA
*Moraxella* spp.	0 (0.0)	1 (0.4)	1 (0.2)	NA
*Ochromobacter* spp.	1 (0.3)	0 (0.0)	1 (0.2)	NA
*Citrobacter* spp.	1 (0.3)	0 (0.0)	1 (0.2)	NA
*Chryseobacterium* spp.	1 (0.3)	0 (0.0)	1 (0.2)	NA
*Aeromonas* spp.	1 (0.3)	0 (0.0)	1 (0.2)	NA
Subtotal	152 (51.2)	154 (62.9)	306 (56.5)	0.007
Total	297 (100.0)	245 (100.0)	542 (100.0)	

**Table 2:** Summary of demographic factors for cases of bacterial keratitis over a 26-year study period.

Demographic Factor	1998–2010 (*n* = 255)	2011–2023 (*n* = 207)	*p*-Value
Age (year)	48.7 ± 21.7	57.3 ± 21.1	<0.001
Male sex	136 (53.3)	116 (56.0)	0.574
Symptom duration (days) *	8.3 ± 23.9	10.1 ± 13.6	0.339
Contact lens wear	55 (21.6)	40 (19.3)	0.565
Diabetes mellitus	21 (8.2)	25 (12.1)	0.211
Prior use of topical antibiotics ^†^	123 (48.2)	103 (49.8)	0.779
Prior use of topical steroids ^‡^	35 (13.7)	32 (15.5)	0.598
Prior ocular surface disease	77 (30.2)	55 (26.6)	0.409
Prior ocular surgery	44 (17.3)	57 (27.5)	0.009
Agricultural occupation	51 (20.0)	57 (27.5)	0.061

* The interval between symptom onset and initial presentation to our hospital. ^†^ The cases in which topical antibiotics were being used at the time of presentation to our hospital, regardless of the duration or type. ^‡^ The cases in which topical steroids were being used at the time of presentation to our hospital, regardless of the duration or type.

**Table 3:** Antibiotic resistance of overall Gram-positive isolates in keratitis.

Antibiotics	Resistant *n*/Tested *n* (%)			*p*-Value
	1998–2010	2011–2023	Total	
Beta-lactams				
Penicillin	120/142 (84.5)	70/88 (79.5)	190/230 (82.6)	0.373
Ampicillin	7/12 (58.3)	15/28 (53.6)	22/40 (55.0)	>0.999
Oxacillin	61/116 (52.6)	28/60 (46.7)	89/176 (50.6)	0.525
Cefotaxime	1/16 (6.3)	1/8 (12.5)	2/24 (8.3)	>0.999
Imipenem	0/6	11/24 (45.8)	11/30 (36.7)	NA
Aminoglycosides				
Amikacin	2/3 (66.7)	1/2 (50.0)	3/5 (60.0)	NA
Gentamicin	61/116 (52.6)	21/61 (34.4)	82/177 (46.3)	0.026
Fluoroquinolones				
Ciprofloxacin	20/66 (30.3)	32/81 (39.5)	52/147 (35.4)	0.299
Norfloxacin	12/21 (57.1)	17/23 (73.9)	29/44 (65.9)	0.342
Levofloxacin	10/25 (40.0)	15/29 (51.7)	25/54 (46.3)	0.425
Moxifloxacin	1/21 (4.8)	2/4 (50.0)	3/25 (12.0)	NA
Subtotal	31/69 (44.9)	40/89 (44.9)	71/158 (44.9)	>0.999
Glycopeptides				
Teicoplanin	6/138 (4.3)	9/81 (11.1)	15/219 (6.8)	0.093
Vancomycin	1/139 (0.7)	7/90 (7.8)	8/229 (3.5)	0.007
Others				
Erythromycin	42/85 (49.4)	38/82 (46.3)	80/167 (47.9)	0.757
Tetracycline	15/39 (38.5)	22/88 (25.0)	37/127 (29.1)	0.142
Tigecycline	0/2	0/80	0/82	NA
Clindamycin	10/29 (34.5)	36/88 (40.9)	46/117 (39.3)	0.662
Linezolid	0/71	0/89	0/160	NA
Synercid	2/31 (6.5)	12/82 (14.6)	14/113 (12.4)	0.343
Rifampicin	2/28 (7.1)	1/59 (1.7)	3/87 (3.4)	0.241
TMP/SMX	45/118 (38.1)	28/87 (32.2)	73/205 (35.6)	0.461
Nitrofurantoin	0/28	13/82 (15.9)	13/110 (11.8)	0.036
Fusidic acid	19/26 (73.1)	37/59 (62.7)	56/85 (65.9)	0.459

TMP/SMX = trimethoprim/sulfamethoxazole.

**Table 4:** Antibiotic resistance of overall Gram-negative isolates in keratitis.

Antibiotics	Resistant *n*/Tested *n* (%)			*p*-Value
	1998–2010	2011–2023	Total	
Beta-lactams				
Ampicillin	69/71 (97.2)	34/42 (81.0)	103/113 (91.2)	0.005
Aztreonam	28/106 (26.4)	69/129 (53.5)	97/235 (41.3)	<0.001
Piperacillin	21/115 (18.3)	32/98 (32.7)	53/213 (24.9)	0.018
Ticarcillin	28/90 (31.1)	9/18 (50.0)	37/108 (34.3)	0.173
Cefoxitin	35/57 (61.4)	27/42 (64.3)	62/99 (62.6)	0.835
Cefotaxime	44/112 (39.3)	69/136 (50.7)	113/248 (45.6)	0.075
Ceftazidime	11/133 (8.3)	12/136 (8.8)	23/269 (8.6)	>0.999
Cefepime	7/125 (5.6)	7/138 (5.1)	14/263 (5.3)	>0.999
Imipenem	4/136 (2.9)	9/138 (6.5)	13/274 (4.7)	0.255
Meropenem	4/115 (3.5)	4/101 (4.0)	8/216 (3.7)	>0.999
Ertapenem	1/1	0/38	1/39 (2.6)	NA
Aminoglycosides				
Amikacin	14/133 (10.5)	2/123 (1.6)	16/256 (6.3)	0.004
Gentamicin	17/120 (14.2)	4/137 (2.9)	21/257 (8.2)	0.001
Tobramycin	15/123 (12.2)	1/24 (4.2)	16/147 (10.9)	0.472
Subtotal	24/135 (17.8)	10/139 (7.2)	34/274 (12.4)	0.010
Fluoroquinolones				
Ciprofloxacin	9/120 (7.5)	19/132 (14.4)	28/252 (11.1)	0.108
Levofloxacin	4/45 (8.9)	1/26 (3.8)	5/71 (7.0)	0.646
Subtotal	10/136 (7.4)	20/140 (14.3)	30/276 (10.9)	0.081
Others				
Tigecycline	-	31/116 (26.7)	31/116 (26.7)	NA
Colistin	4/31 (12.9)	7/95 (7.4)	11/126 (8.7)	0.462
TMP/SMX	35/126 (27.8)	46/149 (30.9)	81/275 (29.5)	0.598

TMP/SMX = trimethoprim/sulfamethoxazole.

## Data Availability

The datasets generated during and/or analyzed during the current study are available from the corresponding author upon reasonable request.

## Supplementary Materials

The following supporting information can be downloaded at https://www.mdpi.com/article/10.3390/antibiotics15020207/s1, Supplementary Table S1. Microbiological profile of bacterial isolates in keratitis between 1998 and 2023. Supplementary Table S2. Antibiotic resistance of representative Gram-positive species. Supplementary Table S3. Antibiotic resistance of representative Gram-negative species.

## Abbreviations

ARMOR	Antimicrobial Resistance Monitoring of Ocular Microorganisms
AST	Antimicrobial susceptibility testing
BLBLI	β-lactam–β-lactamase inhibitor combinations
BK	Bacterial keratitis
CRE	Carbapenem-resistant *Enterobacterales*
ESBL	Extended-spectrum β-lactamase-producing *Enterobacterales*
GNB	Gram-negative bacteria
GPB	Gram-positive bacteria
MDR	Multidrug-resistant
MICs	Minimal inhibitory concentrations
MRAB	MDR *Acinetobacter baumannii*
MRCoNS	Methicillin-resistant coagulase-negative staphylococci
MRCoPS	Methicillin-resistant coagulase-positive staphylococci
MRPA	MDR *Pseudomonas aeruginosa*
MRS	Methicillin-resistant staphylococci
MRSA	Methicillin-resistant *Staphylococcus aureus*
NCCLS	National Committee for Clinical Laboratory Standards
VRE	Vancomycin-resistant *Enterococci*
