# Supplementary material for: Trends of Microorganisms and Antibiotic Resistance Isolated from Patients with Bacterial Keratitis from a Tertiary Hospital in Southeastern Korea: A 26-Year Retrospective Medical Record Review
Source: Antibiotics (Basel). 2026 Feb 13;15(2):207. doi: 10.3390/antibiotics15020207 (PMC12937251; doi:10.3390/antibiotics15020207)
Supplement: Supplementary file 1 [file antibiotics-15-00207-s001.zip › Supplementary file_Table S1_Final.pdf]

Supplementary Table S1. Microbiological profile of bacterial isolates in keratitis between 1998 and 2023.

**Bacterial isolates**	1998–2010 *n* (%)	2011–2023 *n* (%)	Total *n* (%)	**Bacterial isolates**	1998–2010 *n* (%)	2011–2023 *n* (%)	Total *n* (%)
Gram-positive				Gram-negative			
Staphylococcus spp.	115 (38.7)	60 (24.5)	175 (32.3)	*Pseudomonas* spp.	47 (15.8)	51 (20.8)	98 (18.1)
CoPS				*P. aeruginosa*	28 (9.4)	25 (10.2)	53 (9.8)
S. aureus	35 (11.8)	20 (8.2)	55 (10.1)	*P. putida*	6 (2.0)	18 (7.3)	24 (4.4)
CoNS	80 (26.9)	40 (16.3)	120 (22.1)	*P. stutzeri*	3 (1.0)	2 (0.8)	5 (0.9)
S. epidermidis	77 (25.9)	33 (13.5)	110 (20.3)	*P. mendocina*	0 (0)	3 (1.2)	3 (0.6)
S. capitis	1 (0.3)	2 (0.8)	3 (0.6)	*P. fluorescens*	0 (0)	1 (0.4)	1 (0.2)
S. haemolyticus	1 (0.3)	1 (0.4)	2 (0.4)	*P. pickettii*	1 (0.3)	0 (0)	1 (0.2)
S. warneri	0 (0)	2 (0.8)	2 (0.4)	*P.* species	9 (3.0)	2 (0.8)	11 (2.0)
S. hominis	1 (0.3)	0 (0)	1 (0.2)	*Enterobacter* spp.	30 (10.1)	19 (7.8)	49 (9.0)
S. auricularis	0 (0)	1 (0.4)	1 (0.2)	*E. cloacae*	20 (6.7)	15 (6.1)	35 (6.5)
S. kloosii	0 (0)	1 (0.4)	1 (0.2)	*E. aerogenes*	8 (2.7)	4 (1.6)	12 (2.2)
Enterococcus spp.	12 (4.0)	23 (9.4)	35 (6.5)	*E. agglomerans*	2 (0.7)	0 (0)	2 (0.4)
E. faecium	6 (2.0)	11 (4.5)	17 (3.1)	*Serratia* spp.	28 (9.4)	14 (5.7)	42 (7.7)
E. faecalis	6 (2.0)	9 (3.7)	15 (2.8)	*S. marcescens*	27 (9.1)	14 (5.7)	41 (7.6)
E. avium	0 (0)	2 (0.8)	2 (0.4)	*S. liquefaciens*	1 (0.3)	0 (0)	1 (0.2)
E. casseliflavus	0 (0)	1 (0.4)	1 (0.2)	*Stenotrophomonas* spp.			
Streptococcus spp.	18 (6.1)	8 (3.3)	26 (4.8)	*S. maltophilia*	17 (5.7)	15 (6.1)	32 (5.9)
S. pneumoniae	12 (4.0)	2 (0.8)	14 (2.6)	*Acinetobacter* spp.	4 (1.3)	26 (10.6)	30 (5.5)
S. mitis	2 (0.7)	3 (1.2)	5 (0.9)	*A. baumannii*	3 (1.0)	23 (9.4)	26 (4.8)
S. pyogenes	1 (0.3)	1 (0.4)	2 (0.4)	*A. lwoffii*	1 (0.3)	2 (0.8)	3 (0.6)
S. agalactiae	0 (0)	2 (0.8)	2 (0.4)	*A. pittii*	0 (0)	1 (0.4)	1 (0.2)
S. dysgalactiae	1 (0.3)	0 (0)	1 (0.2)	*Achromobacter* spp.	4 (1.3)	16 (6.5)	20 (3.7)
S. sangius	1 (0.3)	0 (0)	1 (0.2)	*A. xylosoxidans*	3 (1.0)	7 (2.9)	10 (1.8)
S. species	1 (0.3)	0 (0)	1 (0.2)	*A. denitrificans*	1 (0.3)	9 (3.7)	10 (1.8)
				Klebsiella spp.	8 (2.7)	1 (0.4)	9 (1.7)
				K. pneumoniae	4 (1.3)	0 (0)	4 (0.7)
				K. oxytoca	4 (1.3)	0 (0)	4 (0.7)
				K. planticola	0 (0)	1 (0.4)	1 (0.2)
				Leclercia spp.			
				L. adecarboxylata	0 (0)	8 (3.3)	8 (1.5)
				Escherichia spp.			
				E. coli	3 (1.0)	2 (0.8)	5 (0.9)
				Delftia spp.			
				D. acidovorans	3 (1.0)	0 (0)	3 (0.6)
				Proteus spp.	1 (0.3)	1 (0.4)	2 (0.4)
				P. vulgaris	0 (0)	1 (0.4)	1 (0.2)
				P. mirabilis	1 (0.3)	0 (0)	1 (0.2)
				Morganella spp.			
				M. morganii	2 (0.7)	0 (0)	2 (0.4)
				Pantoea spp.	1 (0.3)	0 (0)	1 (0.2)
				Moraxella spp.	0 (0)	1 (0.4)	1 (0.2)
				Ochromobacter spp.			
				O. anthropi	1 (0.3)	0 (0)	1 (0.2)
				Citrobacter spp.			
				C. diversus	1 (0.3)	0 (0)	1 (0.2)
				Chryseobacterium spp.			
				C. indologenes	1 (0.3)	0 (0)	1 (0.2)
				Aeromonas spp.	1 (0.3)	0 (0)	1 (0.2)
Subtotal	145 (48.8)	91 (37.1)	236 (43.5)	Subtotal	152 (51.2)	154 (62.9)	306 (56.5)

Percentage (%) is based on number of total isolates in each period (1998–2010, *n* = 297; 2011–2023, *n* = 245, Total period, *n* = 542)
CoNS = Coagulase-negative staphylococci, *CoPS* = Coagulase-positive staphylococci
