# Supplementary material for: Trends of Microorganisms and Antibiotic Resistance Isolated from Patients with Bacterial Keratitis from a Tertiary Hospital in Southeastern Korea: A 26-Year Retrospective Medical Record Review
Source: Antibiotics (Basel). 2026 Feb 13;15(2):207. doi: 10.3390/antibiotics15020207 (PMC12937251; doi:10.3390/antibiotics15020207)
Supplement: Supplementary file 1 [file antibiotics-15-00207-s001.zip › Supplementary file_Table S2_Final.pdf]

Supplementary Table S2. Antibiotic resistance of representative Gram-positive species

**Antibiotics**	*Staphylococcus* spp.			*Enterococcus* spp.			*Streptococcus* spp.		
	Resistant *n* / Tested *n* (%)		*p*-value	Resistant *n* / Tested *n* (%)		*p*-value	Resistant *n* / Tested *n* (%)		*p*-value
	1998–2010	2011–2023		1998–2010	2011–2023		1998–2010	2011–2023	
Beta-lactams									
Penicillin	104/113 (92.0)	53/58 (91.4)	>0.999	9/12 (75)	15/23 (65.2)	0.709	7/17 (41.2)	2/7 (28.6)	0.669
Ampicillin	-	-	-	7/12 (58.3)	13/23 (56.5)	>0.999	-	2/5 (40)	NA
Oxacillin	58/113 (51.3)	28/59 (47.5)	0.748	-	-		3/3	0/1	NA
Cefotaxime	0/2	0/1	NA	-	-		1/14 (7.1)	1/7 (14.3)	>0.999
Imipenem	0/1	-	NA	0/2 (0)	11/23 (47.8)	NA	0/3	0/1	NA
Aminoglycosides									-
Amikacin	1/2 (50)	2/2	NA	-	-		1/1	0/1	>0.999
Gentamicin	60/115 (52.2)	21/60 (35)	0.038	-	-		1/1	0/1	>0.999
Fluoroquinolones									-
Ciprofloxacin	18/63 (28.6)	18/59 (30.5)	0.845	2/2	14/22 (63.6)	NA	0/1	-	NA
Norfloxacin	10/19 (52.6)	-	NA	2/2	17/23 (73.9)	NA	-	-	-
Levofloxacin	8/21 (38.1)	0/1	NA	2/2	12/22 (54.6)	NA	0/2	3/6 (50)	NA
Moxifloxacin	0/19	-	NA	1/1	-	NA	0/1	2/4 (50)	NA
Subtotal	28/64 (43.8)	18/60 (30)	0.138	2/2	17/23 (73.9)	NA	1/3 (33.3)	5/6 (83.3)	0.226
Glycopeptides									-
Teicoplanin	6/111 (5.4)	4/59 (6.8)	0.740	0/23	5/21 (23.8)	0.019	0/15	0/1	NA
Vancomycin	1/110 (0.9)	1/60 (1.7)	>0.999	0/12	6/23 (26.1)	0.074	0/17	0/7	NA
Others									-
Erythromycin	32/61 (52.5)	15/52 (28.9)	0.013	6/7 (85.7)	22/23 (95.7)	0.418	4/17 (23.5)	1/7 (14.3)	>0.999
Tetracycline	4/26 (15.4)	10/59 (16.9)	>0.999	10/11 (90.9)	10/23 (43.5)	0.011	1/2 (50)	2/6 (33.3)	>0.999
Tigecycline	0/2	0/54	NA	-	0/21	NA	-	0/5	NA
Clindamycin	8/27 (29.6)	13/60 (21.7)	0.430	*	*	NA	-	1/6 (16.7)	NA
Linezolid	0/63	0/60	NA	0/7	0/23	NA	0/1	0/6	NA
Synercid	0/28	0/59	NA	2/2	12/23 (52.2)	NA	0/1	-	NA
Rifampicin	2/26 (7.7)	1/58 (1.7)	0.225	-	-	-	0/2	0/1	NA
TMP/SMX	41/113 (36.3)	6/59 (10.2)	<0.001	*	*	NA	2/3 (66.7)	1/5 (20)	NA
Nitrofurantoin	0/26	1/59 (1.7)	>0.999	0/2	12/23 (52.2)	NA	-	-	-
Fusidic acid	19/26 (73.1)	37/59 (62.7)	0.459	-	-	-	-	-	-

TMP/SMX=trimethoprim/sulfamethoxazole.
*Intrinsic resistance
