# Supplementary material for: Trends of Microorganisms and Antibiotic Resistance Isolated from Patients with Bacterial Keratitis from a Tertiary Hospital in Southeastern Korea: A 26-Year Retrospective Medical Record Review
Source: Antibiotics (Basel). 2026 Feb 13;15(2):207. doi: 10.3390/antibiotics15020207 (PMC12937251; doi:10.3390/antibiotics15020207)
Supplement: Supplementary file 1 [file antibiotics-15-00207-s001.zip › Supplementary file_Table S3_Final.pdf]

Supplementary Table S3. Antibiotic resistance of representative Gram-negative species

**Antibiotics**	*Pseudomonas* spp.			*Enterobacter* spp.			*Serratia* spp.			*Acinetobacter* spp.		
	Resistant *n* / Tested *n* (%)		*p*-value	Resistant *n* / Tested *n* (%)		*p*-value	Resistant *n* / Tested *n* (%)		*p*-value	Resistant *n* / Tested *n* (%)		*p*-value
	1998–2010	2011–2023		1998–2010	2011–2023		1998–2010	2011–2023		1998–2010	2011–2023	
Beta-lactams												
Ampicillin	*	*	NA	*	*	NA	*	*	NA	-	-	-
Aztreonam	16/38 (42.1)	36/51 (70.6)	0.009	2/26 (7.7)	0/19	0.501	1/19 (5.3)	0/14	>0.999	3/4 (75)	17/17	NA
Piperacillin	4/40 (10)	7/51 (13.7)	0.750	4/27 (14.8)	1/4 (25)	0.525	0/21	0/2	NA	3/4 (75)	21/26 (80.8)	NA
Ticarcillin	13/47 (27.7)	9/14 (64.3)	0.024	0/4	-	NA	1/17 (6.3)	0/1	NA	2/4 (50)	0/1	NA
Cefoxitin	1/1	-	NA	26/26	17/18 (94.4)	0.409	6/19 (31.6)	9/13 (69.2)	0.070	-	-	
Cefotaxime	28/37 (75.7)	45/51 (88.2)	0.155	1/27 (3.7)	0/18	>0.999	5/22 (22.7)	0/14	0.134	3/4 (75)	9/24 (37.5)	NA
Ceftazidime	5/47 (10.6)	2/51 (3.9)	0.255	0/28	0/18	NA	1/28 (3.6)	0/14	>0.999	1/4 (25)	7/24 (29.2)	NA
Cefepime	2/42 (4.8)	1/51 (2.0)	0.587	0/28	0/18	NA	0/25	0/14	NA	2/4 (50)	1/26 (3.8)	NA
Imipenem	1/47 (2.1)	4/51 (7.8)	0.364	0/29	1/19 (5.3)	0.396	0/28	2/14 (14.3)	0.106	0/4	1/26 (3.8)	NA
Meropenem	2/39 (5.1)	2/51 (3.9)	>0.999	0/26	0/4	NA	0/23	0/2	NA	0/4	1/26 (3.8)	NA
Ertapenem	*	*	NA	-	0/15	NA	-	0/12	NA	*	*	NA
Aminoglycosides												-
Amikacin	5/47 (10.6)	0/50	0.024	0/28	0/18	NA	0/28	0/13	NA	1/4 (25)	0/14	NA
Gentamicin	7/47 (14.9)	1/51 (2.0)	0.026	0/26	0/18	NA	0/22	0/14	NA	1/4 (25)	1/26 (3.8)	NA
Tobramycin	4/37 (10.8)	0/16	0.303	0/28	0/3	NA	2/28 (7.1)	0/2	NA	1/4 (25)	0/1	NA
Netilmicin	3/14 (21.4)	2/5 (40)	0.570	-	-		0/1	-	NA	0/2	-	NA
Fluoroquinolones												
Ciprofloxacin	2/42 (4.8)	3/51 (5.9)	>0.999	0/24	0/15	NA	1/24 (4.2)	0/13	>0.999	2/4 (50)	3/24 (12.5)	NA
Levofloxacin	2/20 (10)	1/11 (9.1)	>0.999	0/6	0/3	NA	0/3	0/2	NA	1/2 (50)	0/2	NA
Subtotal	2/42 (4.8)	3/51 (5.9)	>0.999	0/30	1/19 (5.3)	0.388	1/26 (3.8)	0/14	>0.999	2/4 (50)	3/24 (12.5)	NA
Others												
Tigecycline	*	*	NA	-	0/15	NA	-	0/12	NA	-	1/25 (4.0)	NA
Colistin	0/20	3/49 (6.1)	0.551	0/1	0/1	NA	*	*	NA	0/2	1/26 (3.8)	NA
TMP/SMX	*	*	NA	0/28	0/19	NA	3/21 (14.3)	0/14	0.259	0/4	2/25 (8.0)	NA

TMP/SMX=trimethoprim/sulfamethoxazole.
*Intrinsic resistance
